# Genomic features, evolutionary patterns and minimal residual disease at surgical margins as novel prognostic/predictive biomarkers in locally advanced rectal cancer

**DOI:** 10.1002/ctm2.1286

**Published:** 2023-06-06

**Authors:** Kewen He, Li Li, Aijie Li, Yang Xu, Jiaohui Pang, Dianbin Mu, Jie Ma, Hong Ge, Aurian Maleki, Xueting Qin, Xian Zhang, Qiuxiang Ou, Yang Shao, Jinming Yu, Shuanghu Yuan

**Affiliations:** ^1^ Department of Radiation Oncology Shandong First Medical University and Shandong Academy of Medical Sciences Shandong Cancer Hospital and Institute Jinan China; ^2^ Shanghe County People's Hospital Jinan China; ^3^ Geneseeq Research Institute Nanjing Geneseeq Technology Inc. Nanjing China; ^4^ Department of Pathology Shandong Cancer Hospital and Institute Shandong First Medical University and Shandong Academy of Medical Sciences Jinan China; ^5^ Department of Pathology The Affiliated Cancer Hospital of Zhengzhou University Zhengzhou China; ^6^ Department of Radiation Oncology The Affiliated Cancer Hospital of Zhengzhou University Zhengzhou China; ^7^ Department of Radiation Oncology The University of Texas MD Anderson Cancer Center Houston Texas USA; ^8^ Department of Chemistry Rice University Houston Texas USA; ^9^ School of Medicine Nankai University Tianjin China; ^10^ Department of Bio‐therapeutic the First Medical Center Chinese PLA General Hospital Beijing China; ^11^ School of Public Health Nanjing Medical University Nanjing China

Dear Editor,

Locally advanced rectal cancers (LARCs) are characterized by stage II/III diseases and account for about 15% of colorectal cancer cases.^1^ Currently, a comprehensive trimodality approach of neoadjuvant chemoradiotherapy (nCRT), total mesorectal excision (TME) and adjuvant chemotherapy is the standard of care for LARC.^2^ Previous studies have shown that nCRT could help control locoregional recurrence and improve pathologic complete response rate.^3^, ^4^ Nevertheless, up to 30% of patients showed poor response to nCRT, and as high as 35% of patients suffered from systemic recurrence.^5^, ^6^ This study aimed to use comprehensive genomic profiling to identify reliable molecular biomarkers to stratify LARC patients based on their treatment response, recurrence risk and prognosis.

A total of 76 LARC patients who received nCRT plus TME were included in the analyses (Figure S1A). All patients had baseline tissue biopsies (T0) collected before treatments; after nCRT and TME, 72 patients had matched surgical tumours (T1), whereas 55 had paired surgical margin samples (T2), all of which were classified as tumour‐negative by histological methods (Figure S1B). For all 76 patients, the median age was 56.5, and more patients were males (57, 75.0%) (Table S1). The majority of the patients had stage III tumours (65, 85.5%) based on the pre‐treatment clinical TNM assessment, and the nCRT‐induced downstaging was demonstrated by the corresponding pathological TNM (pTNM) stage. The efficacy of nCRT was assessed using tumour regression grade (TRG),^7^ with TRG0 representing complete tumour regression and TRG3 representing little tumour regression.

Nerve invasion was significantly correlated with TRG using univariate logistic regression (Table S2). Similarly, various baseline genetic alterations, including *KRAS* mutations and amplification of *NFKBIA*, *MYC*, *BCL3* and *ZNF217*, were more enriched in TRG3 than TRG0‐2 patients (Table S3 and Figure 1A). The TRG3 group had a significantly higher chromosomal instability score (CIS), but not the number of non‐synonymous mutations (Figure 1B,C). By multivariate analyses, nerve invasion, *MYC* amplification and *KRAS* mutations were significantly associated with poor nCRT response (Figure 1D). Given previous controversial results regarding *KRAS* and *MYC* alterations,^8^, ^9^, ^10^ we further stratified patients based on the dose of radiation during nCRT. Intriguingly, for patients with *MYC* amplification, there were more completely responded patients (i.e. TRG0) and less poorly responded patients (i.e. TRG3) in the high‐dose (≥50.4 Gy) than the low‐dose (< 50.4 Gy) group (Figure 1E). Similarly, less proportion of *KRAS*‐positive patients would be classified as TRG3 when using high doses of radiation (Figure 1E). We also analysed immune infiltration using 125 rectal cancer patients from the TCGA database. Patients with *KRAS* mutations or *MYC* amplification tended to have less total or CD8^+^ T cell infiltration, respectively (Figures S2 and S3A,B), although the results were not statistically significant in our cohort using immunohistochemistry due to limited sample size and ethnicity differences. Overall, baseline *MYC* amplification and *KRAS* mutations were potential predictive biomarkers for nCRT and might be used to direct the dose of radiation during nCRT.

**FIGURE 1:**
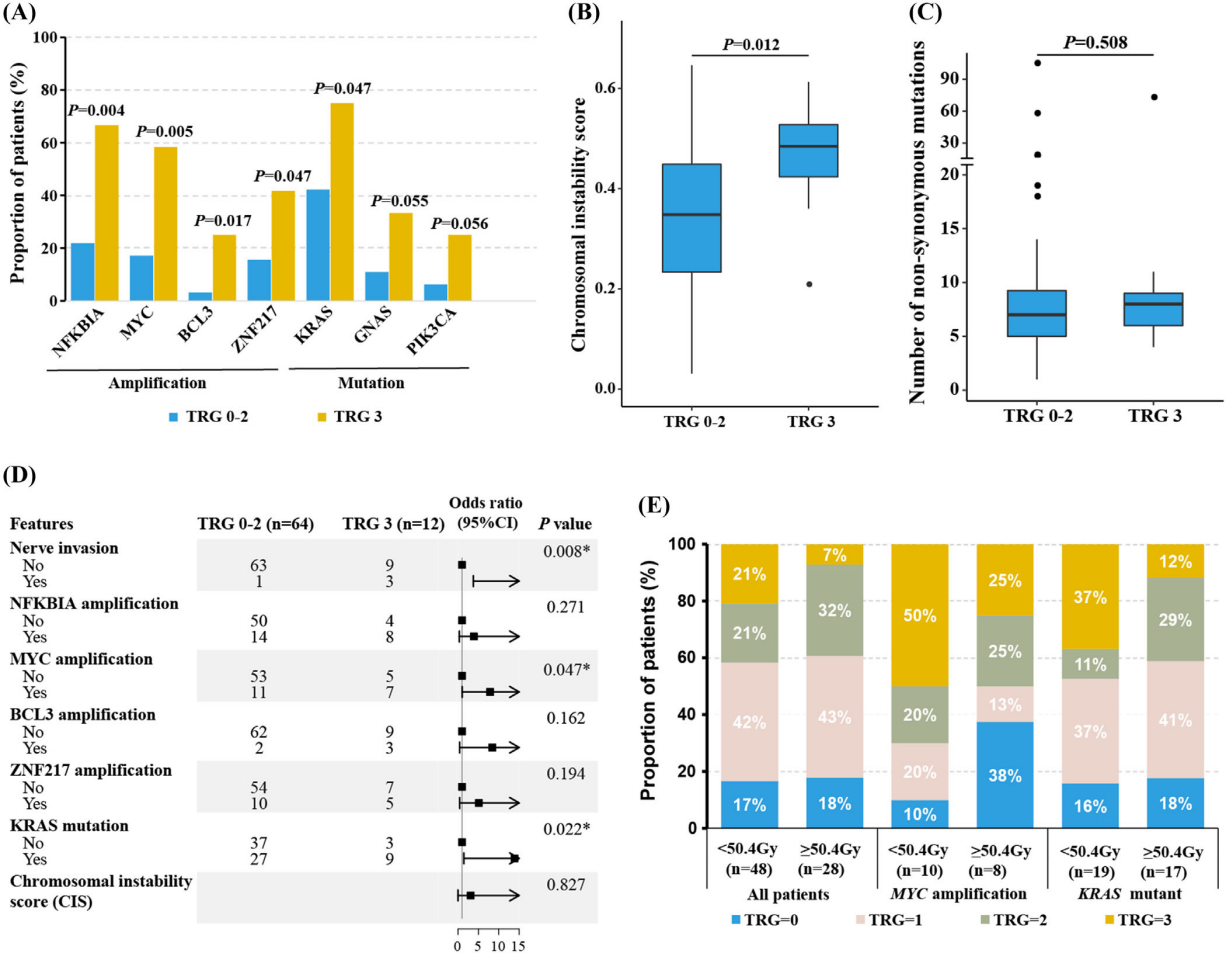
Baseline KRAS mutation and MYC amplification were independently associated with nCRT efficacy. (A) The genetic alterations that were higher in TRG3 than TRG0‐2 patients. (B,C) The chromosomal instability score (B) and number of non‐synonymous mutations (C) between TRG0‐2 and TRG3 groups. (D) The forest plot of multivariate logistic regression analysis using factors that were significant in univariate logistic regression analysis. (E) The proportion of patients with different TRGs stratified by MYC/KRAS status and dose of radiation during nCRT. *p value < .05. Abbreviation: nCRT, neoadjuvant chemoradiotherapy.

Next, we analysed nCRT‐induced genomic changes (*n* = 72 patients, Figure 2A), and 15 (20.8%) patients had no detectable mutations after nCRT. Thirty‐one (43.1%) patients acquired new somatic mutations, seven (9.7%) had an increased number of detected mutations and 24 (33.3%) exhibited increased chromosomal instability post nCRT. The number of non‐synonymous mutations was significantly decreased after nCRT for TRG0‐2 patients, but not for TRG3 patients (Figure 2B). In contrast, TGR3 patients had the most significant decrease in CIS (Figure 2C). Patients with lower grades of TRG were more likely to have their mutations cleared (*p* = .025; Figure 2D), and lower grades of TRG patients were less likely to have an increased number of somatic mutations (*p* = .017; Figure 2E) and acquired mutations (*p* = .009; Figure 2F).

**FIGURE 2:**
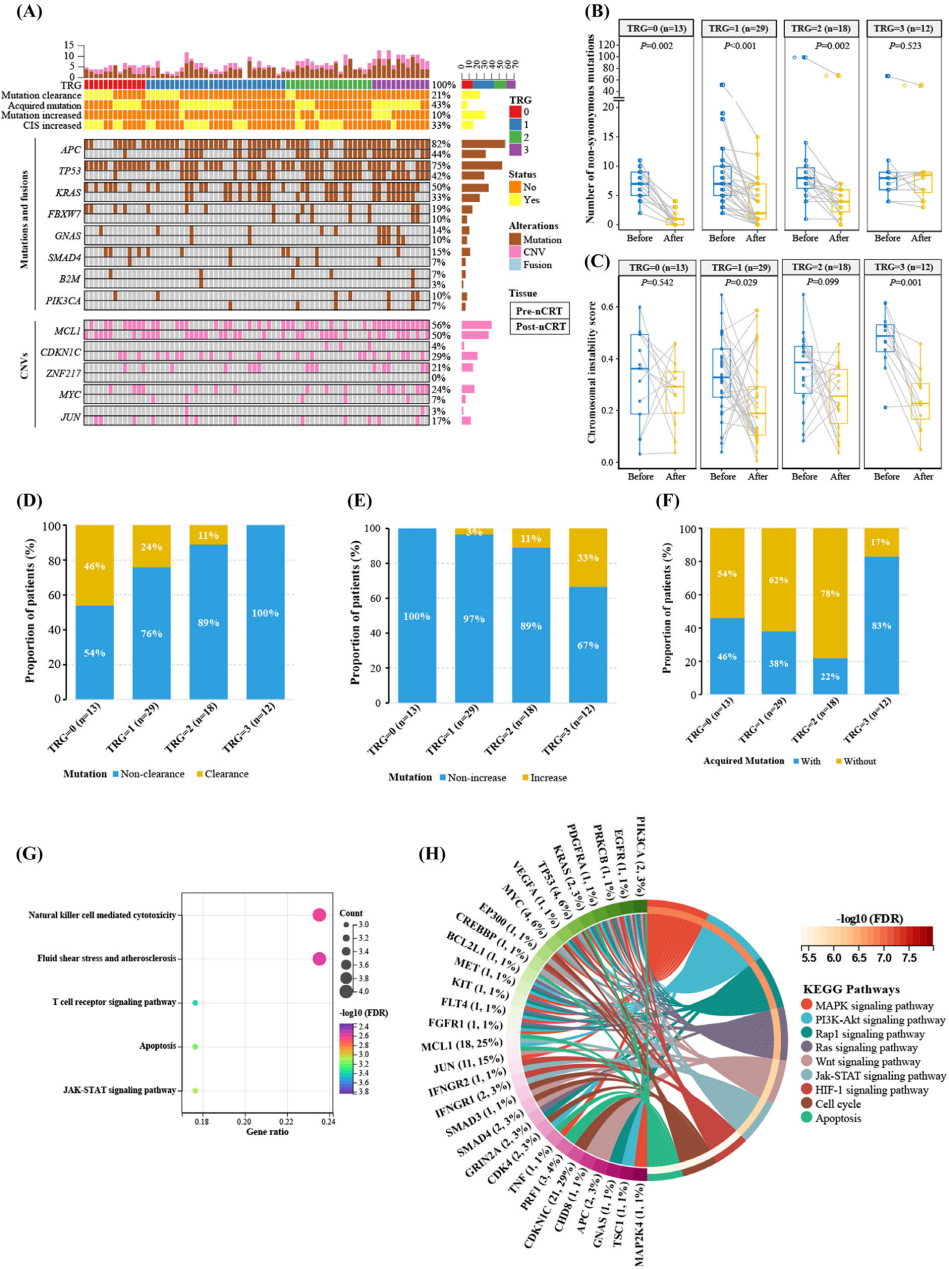
The relationship between nCRT‐induced genomic changes and treatment efficacy. (A) The oncoprint plot of mutations and copy number variants (CNV) of the paired baseline tissue biopsies (the upper row of each gene) and surgical tumour samples (the lower row of each gene) in 72 LARC patients. (B,C) The number of non‐synonymous mutations (B) and chromosomal instability score (C) in TRG0‐3 patients. (D–F) The proportion of cases in TRG0‐3 patients with different post‐nCRT mutation clearance status (D), mutation number changes (E) or status of acquired mutations (F). (G) KEGG pathway analysis using 18 mutated genes that had increased mutational frequency after nCRT treatment. (H) KEGG pathway analysis using the acquired mutated genes after nCRT treatment. Abbreviations: LARC, locally advanced rectal cancer; nCRT, neoadjuvant chemoradiotherapy.

By performing pathway analysis on genes with elevated post‐nCRT mutational frequency (Table S4), five signalling pathways were significantly enriched (Figure 2G). When analysing all pathway‐related mutations detected in the pre‐ and post‐nCRT samples, the five altered pathways were more enriched in pre‐nCRT than post‐nCRT patients (Table S5). Intriguingly, compared with TRG0‐2, the TRG3 group had significantly more aberrations in the T cell receptor, JAK‐STAT and natural killer cell‐mediated cytotoxicity pathways (Table S5). Similarly, by analysing acquired mutations after nCRT (Figure 2H), the RAS and JAK‐STAT pathways were significantly enriched in TRG3 than in TRG0‐2 patients (Table S6). Therefore, multiple pathway alterations, especially JAK‐STAT, might contribute to nCRT resistance.

Based on genetic changes by comparing pre‐ and post‐nCRT (T0 vs. T1) specimens, we grouped them into three evolutionary patterns (Figure 3A): (1) linear evolution with all T1 mutations derived from T0 (Group 1; *n* = 43); (2) parallel evolution with T0 and T1 having completely different mutational profiles (Group 2; *n* = 8); (3) branched evolution with both shared and unique mutations between T0 and T1 (Group 3; *n* = 21). Intriguingly, the proportion of Group 2 patients gradually decreased with increasing TRG grade, whereas TRG3 was specifically enriched with the Group 3 pattern (Figure 3B). Groups 2 and 3 patients had the best and worst disease‐free survival (DFS), respectively (Figure 3C), suggesting that the branched evolutionary pattern was likely to relate to poor nCRT responses and high recurrence risks.

**FIGURE 3:**
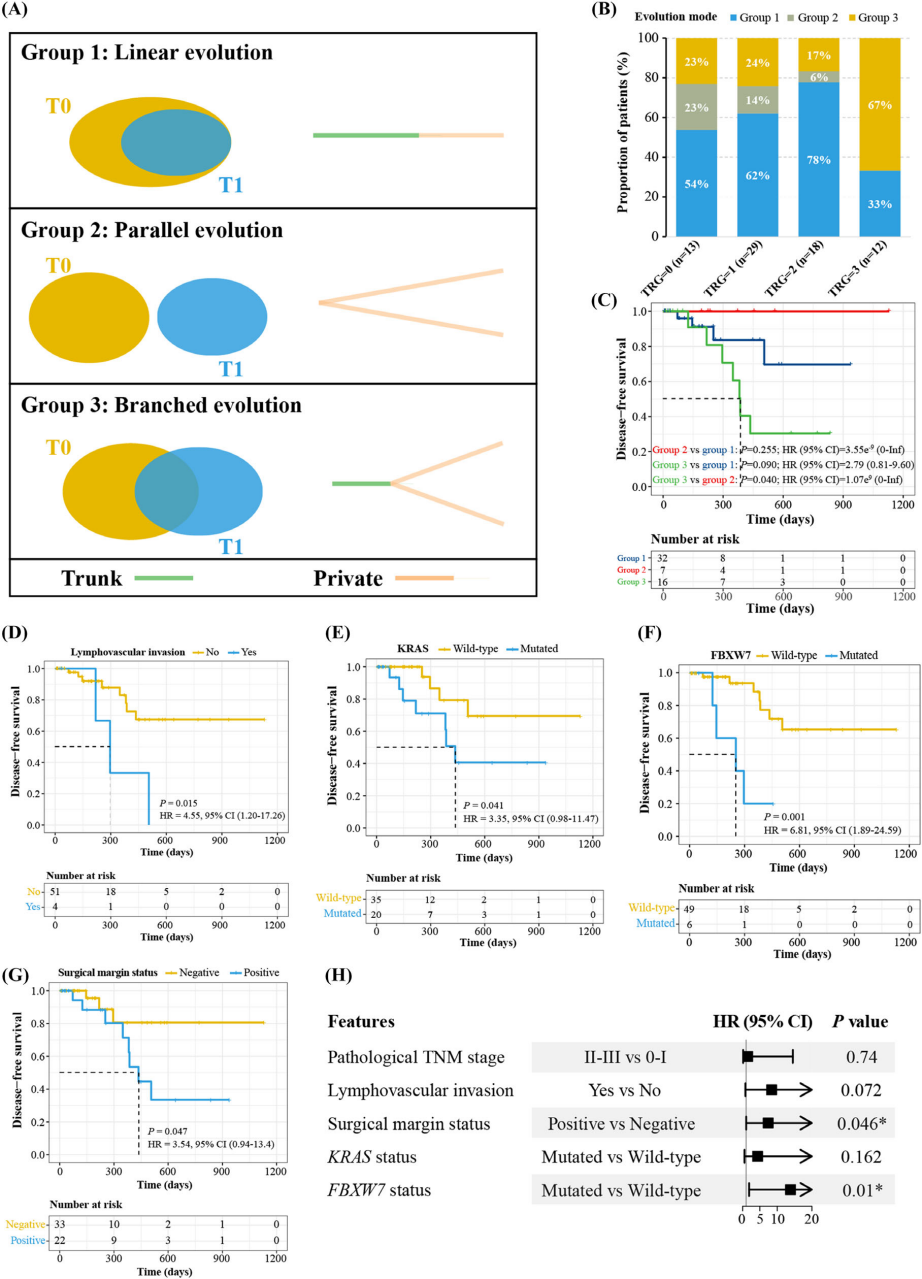
The biomarkers that were associated with post‐surgical recurrence risk. (A) The three tumour evolutionary patterns before and after nCRT. Trunk mutations were those shared between T0 and T1 samples, while private mutations were unique to T0 or T1 tumours. (B) The proportion of cases in TRG0‐3 patients with different evolutionary patterns. (C) Kaplan–Meier curve of disease‐free survival in 55 LARC patients in strata of different evolutionary patterns. (D–G) Kaplan–Meier curve of disease‐free survival in 55 LARC patients in strata of lymphovascular invasion (D), KRAS mutation (E), FBXW7 mutation (F) or surgical margin (G) status. (H) The forest plot of multivariate COX regression analysis using features that were significantly associated with disease‐free survival. *p value < .05. Abbreviations: LARC, locally advanced rectal cancer; nCRT, neoadjuvant chemoradiotherapy.

Lastly, we explored 55 patients with paired surgical tumour (T1) and margin (T2) samples. Lymphovascular invasion and pTNM stage were significantly correlated with disease recurrence (Figures 3D, S4 and Table S7), and *KRAS* or *FBXW7* mutations in T1 were significantly associated with worse DFS (Figure 3E,F). Despite all 55 patients with histologically negative surgical margins, 22 (40.0%) were positive for residual tumours at surgical margins based on next generation sequencing (NGS) assessment. Moreover, surgical margin‐positive patients were more likely to have higher TRG grades (*p* = .047; Table S8) and poorer DFS (Figure 3G). Using multivariate analyses, surgical margin and *FBXW7* mutation were significantly correlated with post‐surgical recurrence risk (Figure 3H).

Overall, we found that baseline *KRAS* mutation and *MYC* amplification might serve as predictive biomarkers for nCRT, and NGS‐based surgical margin status and the nCRT‐induced evolutionary pattern were associated with both nCRT efficacy and post‐surgical recurrence risk. Our results suggest that these molecular features could facilitate the estimation of patient prognosis, direct nCRT regimen and stratify patients for more intense adjuvant therapies.

## FUNDING INFORMATION

National Natural Science Foundation of China (Grant No. NSFC82073345), Natural Science Foundation of Shandong Province Innovation and Development Joint Fund (ZR202209010002), and the Taishan Scholars Program and Jinan Clinical Medicine Science and Technology Innovation Plan (202019060) to Shuanghu Yuan, as well as the Academic Promotion Program of Shandong First Medical University (2019ZL002), Research Unit of Radiation Oncology, Chinese Academy of Medical Sciences (2019RU071), National Natural Science Foundation of China (81627901, 81972863, 82030082 and 31900649), Natural Science Foundation of Shandong (ZR201911040452 and ZR2019LZL018), Cancer Prevention and Treatment Fund of Natural Science Foundation of Shandong Province (ZR2020LZL014) to Jinming Yu.

## CONFLICT OF INTEREST STATEMENT

Jiaohui Pang, Yang Xu, Xian Zhang, Qiuxiang Ou and Yang Shao are staff of Nanjing Geneseeq Technology Inc. The rest of the authors disclosed no conflict of interest.

## ETHICS APPROVAL AND CONSENT TO PARTICIPATE

This study was approved by the institutional research ethics committees of Shandong Cancer Hospital and Institute and Henan Cancer Hospital (ethical number: SDZLEC2018‐043‐01). All patients provided written informed consent to participate and publication, and written informed consent was received prior to participation.

## Supporting information

Supporting Information
