# Supplementary material for: Genomic features, evolutionary patterns and minimal residual disease at surgical margins as novel prognostic/predictive biomarkers in locally advanced rectal cancer
Source: Clin Transl Med. 2023 Jun 6;13(6):e1286. doi: 10.1002/ctm2.1286 (PMC10246683; doi:10.1002/ctm2.1286)
Supplement: Supplementary file 1 — Supporting Information [file CTM2-13-e1286-s001.docx]

## **Materials and Methods**

## **Patients and study design**

This study was approved by the institutional research ethics committees of Shandong Cancer Hospital and Institute and Henan Cancer Hospital (ethical number: SDZLEC2018-043-01). All patients provided written informed consent to participate and publication, and written informed consent was received prior to participation. A total of 105 LARC patients (cT_3-4_N+M_0_) were diagnosed and treated in Shandong Cancer Hospital and Institute and Henan Cancer Hospital from January 2016 to December 2019. Baseline tumor samples from 16 patients did not pass the sequencing quality control, 6 patients did not have detectable baseline somatic mutations, 6 patients had no available clinical information, and 1 patient did not receive neoadjuvant treatment. These 29 patients were thus excluded from the study, and the remaining 76 LARC patients were included for further analyses. Baseline tumor biopsy samples (i.e., prior to any treatments; n=76) and the matched surgical samples (75 surgical tumor samples and 61 surgical margin samples) were subjected to NGS of 474 cancer-relevant genes, including genes related to the efficacy and toxicity of radiotherapy/chemotherapy, the target genes of classic targeted therapies, the immunotherapy-associated genes, as well as genes related to tumorigenesis and familial cancers. The surgical margin samples were collected at least 3 cm away from the tumor. Of note, all the patients received complete surgical resection (i.e., R0 resection) and all the surgical margins were classified as tumor negative by hematoxylin and eosin (H&E) staining. Tumor regression grade (TRG) was evaluated according to the 8^th^ American Joint Committee on Cancer (AJCC) Staging Manual (TRG0: no residual tumor cells; TRG1: single cell or small group of cells; TRG2: residual cancer with desmoplastic responses; TRG3: minimal evidence of tumor response).^1^

## **DNA extraction and library preparation**

Formalin-fixed paraffin-embedded (FFPE) tumor and resection margin samples were de-paraffinized with xylene, and genomic DNA was extracted using QIAamp DNA FFPE Tissue Kit (Qiagen). Purified genomic DNA was qualified by Nanodrop2000 for A260/280 and A260/A230 ratios (Thermo Fisher Scientific). All DNA samples were quantified by Qubit 3.0 using the dsDNA HS Assay Kit (Life Technologies) according to the manufacturer’s recommendations.

Sequencing libraries were prepared using the KAPA Hyper Prep kit (KAPA Biosystems, Wilmington, MA) with an optimized manufacturer’s protocol.^2^ Briefly, ∼1-2 μg of DNA was sequentially undergone end-repairing, A-tailing, and ligation with indexed adapters, followed by size selection using Agencourt AMPure XP beads (Beckman Coulter, Mississauga, Canada) and PCR amplification using KAPA Hyper DNA Library Prep Kit (KAPA Biosystems, Wilmington, MA). Target enrichment was performed using customized xGen lockdown probes (Integrated DNA Technologies) targeting 474 cancer-relevant genes (Radiotron®, Nanjing Geneseeq Technology Inc., Nanjing). The hybridization capture reaction was performed with Dynabeads M-279 (Life Technologies) and xGen Lockdown hybridization and wash kit (Integrated DNA Technologies) according to the manufacturer’s protocols. Captured libraries were on-beads PCR amplified with Illumina p5 and p7 primers in KAPA HiFi HotStart ReadyMix (KAPA Biosystems), followed by purification using Agencourt AMPure XP beads. Libraries were quantified by qPCR using the KAPA Library Quantification kit (KAPA Biosystems). Library fragment size was determined by Bioanalyzer 2100 (Agilent Technologies).

## **Next-generation sequencing and data processing**

Sequencing was performed on the Illumina HiSeq4000 platform followed by data analysis as previously described.^2, 3^ In brief, sequencing data were analyzed by Trimmomatic^4^ to remove low-quality (quality < 15) or N bases, and then mapped to the human reference genome hg19 using the Burrows-Wheeler Aligner (https://github.com/lh3/bwa/tree/master/bwakit). PCR duplicates were removed by Picard (available at: https://broadinstitute.github.io/picard/). The Genome Analysis Toolkit (GATK) (https://software.broadinstitute.org/gatk/) was used to perform local realignments around indels and base quality reassurance. Single nucleotide polymorphisms (SNPs) and indels were analyzed by VarScan2^5^ and Haplotype Caller/UniedGenotyper in GATK. Common SNPs were excluded if they were present in > 1% population frequency in the 1000 Genomes Project or the Exome Aggregation Consortium (ExAC) 65,000 exomes database. Gene fusions were identified by FACTERA.^6^

The cut-offs of variant allele frequency (VAF) and sequencing reads/depth for various genetic changes were as follows: VAF ≥ 0.5%, supporting reads ≥ 3, and depth ≥ 30X for recurrent variants (≥20 mentions in COSMIC v92); VAF ≥ 1%, supporting reads ≥ 6, and depth ≥ 30X for non-recurrent variants; split-reads ≥ 3 for structural variant (SV); copy number variants (CNVs) were hotspots in TCGA database^7^ with gene ratio≥2 as copy number gain and gene ratio <0.6 as copy number loss. If a mutation was detected from the tumor biopsy or the surgical tumor sample by the above criteria, other samples from the same patient would be classified as positive for this mutation for the following cut-offs: recurrent variants were VAF ≥ 0.5% or supporting reads ≥ 2 and depth ≥ 30X; non-recurrent variants were VAF ≥ 0.5% or supporting reads ≥ 4 and depth ≥ 30X. Chromosomal instability score (CIS) was defined as the proportion of the genome with aberrant (purity-adjusted segment-level copy number with log 2 ratio ≥0.2 or ≤-0.2) segmented copy number.^8^

## **Immune infiltration analysis**

The DNA-sequencing data of 125 stage I-III rectal cancer patients were downloaded from cBioPortal (<http://www.cbioportal.org/>). The infiltrated immune cell data were downloaded from TIMER (Tumor IMmune Estimation Resource), which was analyzed using the matched RNA-sequencing data (<https://cistrome.shinyapps.io/timer/_w_9d3e7fa7/immuneEstimation.txt>).

**Immunohistochemistry (IHC)**

The samples were fixed with 4% paraformaldehyde, and trimming, dehydration, embedding, slicing, staining, and sealing were carried out in strict accordance with the pathological experiment inspection procedures. The qualified samples were examined by microscope. Image-Pro Plus 6.0 analysis software was used to analyze the imaging data. Specifically, the number of positive cells and the area of ​​the tissue in each picture were calculated (the number of positive cells per unit area = the number of positive cells / tissue area).

## **Statistical analysis**

Disease-free survival (DFS) refers to the time from the date of curative surgery to tumor relapse/patient death. The pathway of selected genes was analyzed using Kyoto Encyclopedia of Genes and Genomes (KEGG) pathway enrichment analysis. Kaplan-Meier survival curve was used to analyze the DFS of various patient groups, and the statistical difference was analyzed using the log‐rank test. Univariate and multivariable analyses were performed using either logistic regression (for TRG-related analysis) or Cox proportional hazards regression (for DFS-related analysis). The paired samples were analyzed using the Wilcoxon test. Comparisons of the proportion between groups were done using Fisher’s exact test. Multiple comparisons were adjusted using the false discovery rate (FDR). Statistical analyses were performed using the R (v4.2.0), and a two-sided *P* value of <0.05 was considered to be statistically significant.

**Reference**

1. Amin MB, Greene FL, Edge SB*, et al.* The Eighth Edition AJCC Cancer Staging Manual: Continuing to build a bridge from a population-based to a more "personalized" approach to cancer staging. *CA Cancer J Clin* 2017; **67**: 93-99.

2. Yang Z, Yang N, Ou Q*, et al.* Investigating Novel Resistance Mechanisms to Third-Generation EGFR Tyrosine Kinase Inhibitor Osimertinib in Non-Small Cell Lung Cancer Patients. *Clin Cancer Res* 2018; **24**: 3097-3107.

3. Shu Y, Wu X, Tong X*, et al.* Circulating Tumor DNA Mutation Profiling by Targeted Next Generation Sequencing Provides Guidance for Personalized Treatments in Multiple Cancer Types. *Sci Rep* 2017; **7**: 583.

4. Bolger AM, Lohse M, Usadel B. Trimmomatic: a flexible trimmer for Illumina sequence data. *Bioinformatics* 2014; **30**: 2114-2120.

5. Koboldt DC, Zhang Q, Larson DE*, et al.* VarScan 2: somatic mutation and copy number alteration discovery in cancer by exome sequencing. *Genome Res* 2012; **22**: 568-576.

6. Newman AM, Bratman SV, Stehr H*, et al.* FACTERA: a practical method for the discovery of genomic rearrangements at breakpoint resolution. *Bioinformatics* 2014; **30**: 3390-3393.

7. Sanchez-Vega F, Mina M, Armenia J*, et al.* Oncogenic Signaling Pathways in The Cancer Genome Atlas. *Cell* 2018; **173**: 321-337 e310.

8. Zhang X, Liu F, Bao H*, et al.* Distinct genomic profile in h. pylori-associated gastric cancer. *Cancer Med* 2021.

**Supplementary tables and figures**

**Table S1** Demographic and clinical characteristics of the 76 LARC patients.

| **Characteristics** | **All patients (n=76)** |
| --- | --- |
| Median follow-up (days); median (95% CI) | 262 (196-508)^#^ |
| Median age (years); median (range) | 56.5 (14~80) |
| Sex, n (%) |  |
| Female | 19 (25.0%) |
| Male | 57 (75.0%) |
| Clinical TNM stage, n (%) |  |
| II | 11 (14.5%) |
| III | 65 (85.5%) |
| Pathological TNM stage, n (%) |  |
| 0 | 13 (17.1%) |
| I | 19 (25.0%) |
| II | 34 (44.7%) |
| III | 10 (13.2%) |
| Tumor regression grade (TRG), n (%) |  |
| 0 | 13 (17.1%) |
| 1 | 32 (42.1%) |
| 2 | 19 (25.0%) |
| 3 | 12 (15.8%) |
| Neoadjuvant chemoradiotherapy, n (%) |  |
| Concurrent | 74 (97.4%) |
| Sequential | 2 (2.6%) |
| Adjuvant chemotherapy, n (%) |  |
| Yes | 55 (72.4%) |
| No | 3 (3.9%) |
| Unknown | 18 (23.7%) |
| Lymphovascular invasion, n (%) |  |
| Yes | 5 (6.6%) |
| No | 71 (93.4%) |
| Nerve invasion, n (%) |  |
| Yes | 4 (5.3%) |
| No | 72 (94.7%) |
| Recurrence at any site, n (%) |  |
| Yes | 16 (21.1%) |
| No | 56 (73.7%) |
| Unknown | 4 (5.3%) |
| Number of examined lymph nodes; median (range) | 8 (0~20) |
| Number of positive lymph nodes; median (range) | 0 (0~14) |
| Metastatic lymph node ratio (MLNR); median (range) | 0 (0~93.3%) |

^#^Four patients were lost to follow-up.

**Table S2** The univariate analysis between clinical features and nCRT efficacy (n=76).

| **Features** | **Feature status** | **TRG 0~2** | **TRG 3** | **Coefficient** | **Odds ratio (95%CI)** | ***P* value** |
| --- | --- | --- | --- | --- | --- | --- |
|  |  | **n=64** | **n=12** |  |  |  |
| Age (median) |  | 56.5 | 56.5 | -0.019 | 0.98 (0.93-1.03) | 0.447 |
| Clinical TNM stage | II | 8 | 3 |  | 1 (reference) | 0.269 |
|  | III | 56 | 9 | -0.847 | 0.43 (0.10-1.92) |  |
| Sex | Female | 17 | 2 |  | 1 (reference) | 0.472 |
|  | Male | 47 | 10 | 0.593 | 1.81 (0.36~ 9.10) |  |
| Radiation dose (Gy) | <50.4 | 38 | 10 |  | 1 (reference) | 0.131 |
|  | ≥50.4 | 26 | 2 | -1.23 | 0.29 (0.06-1.45) |  |
| Lymphovascular invasion | No | 61 | 10 |  | 1 (reference) | 0.15 |
|  | Yes | 3 | 2 | 1.403 | 4.07 (0.60~ 27.47) |  |
| Nerve invasion | No | 63 | 9 |  | 1 (reference) | 0.012* |
|  | Yes | 1 | 3 | 3.045 | 21.00 (1.97~ 224.32) |  |

**P* value<0.05 for univariate analysis (logistic regression).

**Table S3** The univariate analysis between molecular features and nCRT efficacy (n=76).

| **Features** | **Feature status** | **TRG 0-2** | **TRG 3** | **Coefficient** | **Odds ratio (95%CI)** | ***P* value** |
| --- | --- | --- | --- | --- | --- | --- |
|  |  | **n=64** | **n=12** |  |  |  |
| *NFKBIA* amplification | Yes | 14 | 8 | 1.966 | 7.14 (1.87-27.24) | 0.004* |
|  | No | 50 | 4 |  | 1 (reference) |  |
| *MYC* amplification | Yes | 11 | 7 | 1.909 | 6.75(1.8-25.22) | 0.005* |
|  | No | 53 | 5 |  | 1 (reference) |  |
| *BCL3* amplification | Yes | 2 | 3 | 2.335 | 10.33 (1.51-70.55) | 0.017* |
|  | No | 62 | 9 |  | 1 (reference) |  |
| *ZNF217* amplification | Yes | 10 | 5 | 1.35 | 3.86 (1.02-14.6) | 0.047* |
|  | No | 54 | 7 |  | 1 (reference) |  |
| *KRAS* mutation | Yes | 27 | 9 | 1.414 | 4.11 (1.02-16.63) | 0.047* |
|  | No | 37 | 3 |  | 1 (reference) |  |
| *GNAS* mutation | Yes | 7 | 4 | 1.404 | 4.07 (0.97-17.08) | 0.055 |
|  | No | 57 | 8 |  | 1 (reference) |  |
| *PIK3CA* mutation | Yes | 4 | 3 | 1.609 | 5 (0.96-26.11) | 0.056 |
|  | No | 60 | 9 |  | 1 (reference) |  |
| *MET* amplification | Yes | 2 | 2 | 1.825 | 6.2 (0.78-49.17) | 0.084 |
|  | No | 62 | 10 |  | 1 (reference) |  |
| Chromosomal instability score |  | 0.348 | 0.485 | 7.121 | 1238.03 (4.80-319449.25) | 0.012* |
| Number of non-synonymous mutations |  | 7 | 8 | 0.011 | 1.01 (0.98-1.05) | 0.508 |

**P* value<0.05 for univariate analysis (logistic regression).

**Table S4** List of genes whose mutational frequency increased after nCRT.

| **Gene** | **Pre-nCRT (n=72)** | **Post-nCRT (n=72)** | ***P* value** |
| --- | --- | --- | --- |
| *CDKN1C* variant | 3 (4.2%) | 21 (29.2%) | <0.001 |
| *JUN* variant | 3 (4.2%) | 13 (18.1%) | 0.015 |
| *PRF1* variant | 0 (0%) | 3 (4.2%) | 0.245 |
| *IFNGR1* variant | 0 (0%) | 2 (2.8%) | 0.497 |
| *CDK4* variant | 1 (1.4%) | 2 (2.8%) | 1 |
| *CEBPA* variant | 0 (0%) | 1 (1.4%) | 1 |
| *CHD8* variant | 2 (2.8%) | 3 (4.2%) | 1 |
| *ETV1* variant | 0 (0%) | 1 (1.4%) | 1 |
| *FANCD2* variant | 1 (1.4%) | 2 (2.8%) | 1 |
| *FOXL2* variant | 0 (0%) | 1 (1.4%) | 1 |
| *IFNGR2* variant | 0 (0%) | 1 (1.4%) | 1 |
| *KEAP1* variant | 1 (1.4%) | 2 (2.8%) | 1 |
| *NFE2L2* variant | 1 (1.4%) | 2 (2.8%) | 1 |
| *PDGFRA* variant | 1 (1.4%) | 2 (2.8%) | 1 |
| *PRSS1* variant | 0 (0%) | 1 (1.4%) | 1 |
| *TMPRSS2* variant | 0 (0%) | 1 (1.4%) | 1 |
| *TNF* variant | 0 (0%) | 1 (1.4%) | 1 |
| *TTF1* variant | 2 (2.8%) | 3 (4.2%) | 1 |

**Table S5** The relationship between enriched pathways (derived from 18 elevated mutated genes) and nCRT efficacy.

| **Signaling pathways** | **Feature status** | **Pre-nCRT** | **Post-nCRT** | ***P* value** | **TRG 0-2** | **TRG 3** | ***P* value** |
| --- | --- | --- | --- | --- | --- | --- | --- |
|  |  | **n=72** | **n=72** |  | **n=60** | **n=12** |  |
| T cell receptor | Variant | 40 (55.6%) | 35 (48.6%) | 0.505 | 29 (48.3%) | 11 (91.7%) | 0.009* |
|  | Wild-type | 32 (44.4%) | 37 (51.4%) |  | 31 (51.7%) | 1 (8.3%) |  |
| JAK-STAT | Variant | 30 (41.7%) | 13 (18.1%) | 0.003* | 21 (35.0%) | 9 (75.0%) | 0.022* |
|  | Wild-type | 42 (58.3%) | 59 (81.9%) |  | 39 (65.0%) | 3 (25.0%) |  |
| Natural killer cell mediated cytotoxicity | Variant | 39 (54.2%) | 26 (36.1%) | 0.044* | 29 (48.3%) | 10 (83.3%) | 0.031* |
|  | Wild-type | 33 (45.8%) | 46 (63.9%) |  | 31 (51.7%) | 2 (16.7%) |  |
| Fluid shear stress and atherosclerosis | Variant | 57 (79.2%) | 39 (54.2%) | 0.002* | 45 (75.0%) | 12 (100%) | 0.06 |
|  | Wild-type | 15 (20.8%) | 33 (45.8%) |  | 15 (25.0%) | 0 (0%) |  |
| Apoptosis | Variant | 63 (87.5%) | 47 (65.3%) | 0.003* | 51 (85.0%) | 12 (100%) | 0.34 |
|  | Wild-type | 9 (12.5%) | 25 (34.7%) |  | 9 (15.0%) | 0 (0%) |  |

**P* values<0.05.

**Table S6** The relationship between enriched pathways (derived from acquired mutations) and nCRT efficacy.

| **Signaling pathways** | **Feature status** | **Pre-nCRT** | **Post-nCRT** | ***P* value** | **TRG 0-2** | **TRG 3** | ***P* value** |
| --- | --- | --- | --- | --- | --- | --- | --- |
|  |  | **n=72** | **n=72** |  | **n=60** | **n=12** |  |
| MAPK | Variant | 64 (88.9%) | 47 (65.3%) | 0.001* | 52 (86.7%) | 12 (100%) | 0.337 |
|  | Wild-type | 8 (11.1%) | 25 (34.7%) |  | 8 (13.3%) | 0 (0%) |  |
| PI3K-AKT | Variant | 65 (90.3%) | 43 (59.7%) | <0.001* | 53 (88.3%) | 12 (100%) | 0.592 |
|  | Wild-type | 7 (9.7%) | 29 (40.3%) |  | 7 (11.7%) | 0 (0%) |  |
| RAP1 | Variant | 48 (66.7%) | 29 (40.3%) | 0.003* | 37 (61.7%) | 11 (91.7%) | 0.051 |
|  | Wild-type | 24 (33.3%) | 43 (59.7%) |  | 23 (38.3%) | 1 (8.3%) |  |
| RAS | Variant | 46 (63.9%) | 30 (41.7%) | 0.012* | 35 (58.3%) | 11 (91.7%) | 0.045* |
|  | Wild-type | 26 (36.1%) | 42 (58.3%) |  | 25 (41.7%) | 1 (8.3%) |  |
| WNT | Variant | 71 (98.6%) | 49 (68.1%) | <0.001* | 59 (98.3%) | 12 (100%) | 1 |
|  | Wild-type | 1 (1.4%) | 23 (31.9%) |  | 1 (1.7%) | 0 (0%) |  |
| JAK-STAT | Variant | 30 (41.7%) | 13 (18.1%) | 0.003* | 21 (35.0%) | 9 (75.0%) | 0.022* |
|  | Wild-type | 42 (58.3%) | 59 (81.9%) |  | 39 (65.0%) | 3 (25.0%) |  |
| HIF-1 | Variant | 21 (29.2%) | 12 (16.7%) | 0.112 | 17 (28.3%) | 4 (33.3%) | 0.737 |
|  | Wild-type | 51 (70.8%) | 60 (83.3%) |  | 43 (71.7%) | 8 (66.7%) |  |
| Cell cycle | Variant | 61 (84.7%) | 39 (54.2%) | <0.001* | 50 (83.3%) | 11 (91.7%) | 0.677 |
|  | Wild-type | 11 (15.3%) | 33 (45.8%) |  | 10 (16.7%) | 1 (8.3%) |  |
| Apoptosis | Variant | 63 (87.5%) | 47 (65.3%) | 0.003* | 51 (85.0%) | 12 (100%) | 0.34 |
|  | Wild-type | 9 (12.5%) | 25 (34.7%) |  | 9 (15.0%) | 0 (0%) |  |
| Fluid shear stress and atherosclerosis | Variant | 57 (79.2%) | 39 (54.2%) | 0.002* | 45 (75.0%) | 12 (100%) | 0.06 |
|  | Wild-type | 15 (20.8%) | 33 (45.8%) |  | 15 (25.0%) | 0 (0%) |  |

**P* values<0.05.

**Table S7** The univariate COX analysis of various demographic/clinical features and post-surgical recurrence risk.

| **Characteristics** | **Frequency** | **HR (95% CI)** | ***P* value** |
| --- | --- | --- | --- |
| Age (year) |  |  | 0.86 |
| <57 | 25 (45.5%) | 1 (reference) |  |
| ≥57 | 30 (54.5%) | 0.9 (0.27-2.96) |  |
| Sex |  |  | 0.497 |
| Female | 14 (25.5%) | 1 (reference) |  |
| Male | 41 (74.5%) | 0.65 (0.19-2.26) |  |
| Pathological TNM stage |  |  | 0.027* |
| 0-I | 23 (41.8%) | 1 (reference) |  |
| II-III | 32 (58.2%) | 7.24 (0.92-56.60) |  |
| TRG |  |  | 0.303 |
| 0-2 | 45 (81.8%) | 1 (reference) |  |
| 3 | 10 (18.2%) | 1.90 (0.55-6.56) |  |
| nCRT |  |  | 0.088 |
| Sequential | 2 (3.6%) | 1 (reference) |  |
| Concurrent | 53 (96.4%) | 0.17 (0.02-1.69) |  |
| Radiotherapy dose (Gy) |  |  | 0.588 |
| <50.4 | 37 (67.3%) | 1 (reference) |  |
| ≥50.4 | 18 (32.7%) | 1.40 (0.41-4.82) |  |
| Lymphovascular invasion |  |  | 0.015* |
| No | 51 (92.7%) | 1 (reference) |  |
| Yes | 4 (7.3%) | 4.55 (1.20-17.26) |  |
| Nerve invasion |  |  | 0.782 |
| No | 53 (96.4%) | 1 (reference) |  |
| Yes | 2 (3.6%) | 0 (0-Inf) |  |
| Number of examined lymph nodes |  |  | 0.344 |
| <12 | 44 (80%) | 1 (reference) |  |
| ≥12 | 11 (20%) | 1.80 (0.53-6.15) |  |
| Number of positive lymph nodes |  |  | 0.438 |
| =0 | 45 (81.8%) | 1 (reference) |  |
| ≥1 | 10 (18.2%) | 1.68 (0.44-6.37) |  |
| Metastatic lymph node ratio (MLNR) |  |  | 0.892 |
| <0.2 | 50 (90.9%) | 1 (reference) |  |
| ≥0.2 | 5 (9.1%) | 0.87 (0.11-6.79) |  |

**P* values<0.05.

**Table S8** The relationship between surgical margin status and TRG.

| **Mutation of surgical margin** | **TRG=0** | **TRG=1** | **TRG=2** | **TRG=3** | ***P* value** |
| --- | --- | --- | --- | --- | --- |
| **Positive (n=22)** | 2 (25%) | 8 (33%) | 4 (31%) | 8 (80%) | 0.047* |
| **Negative (n=33)** | 6 (75%) | 16 (67%) | 9 (69%) | 2 (20%) |  |

**P* values<0.05.

**Figure S1** The plan and aim of the study. (**A**) The flowchart of the patients and their matched tumor samples that were included in the study. Specifically, 76 LARC patients with baseline tissue biopsies were used to assess nCRT efficacy, 72 patients with paired baseline tissue biopsies and surgical tumor samples were used to analyze the genomic changes induced by nCRT, and 55 patients with paired surgical tumor and surgical margin samples were used to assess the post-surgical recurrence risk. (**B**) The schematic of sample collection during the whole treatment process.

**Figure S2** Immune infiltration was significantly associated with the clinical outcome in rectal cancer patients. Kaplan-Meier curve of disease-free survival in stage I-III rectal patients in strata of the level of T cell infiltration (**A**) or CD8+ T cell infiltration (**B**).


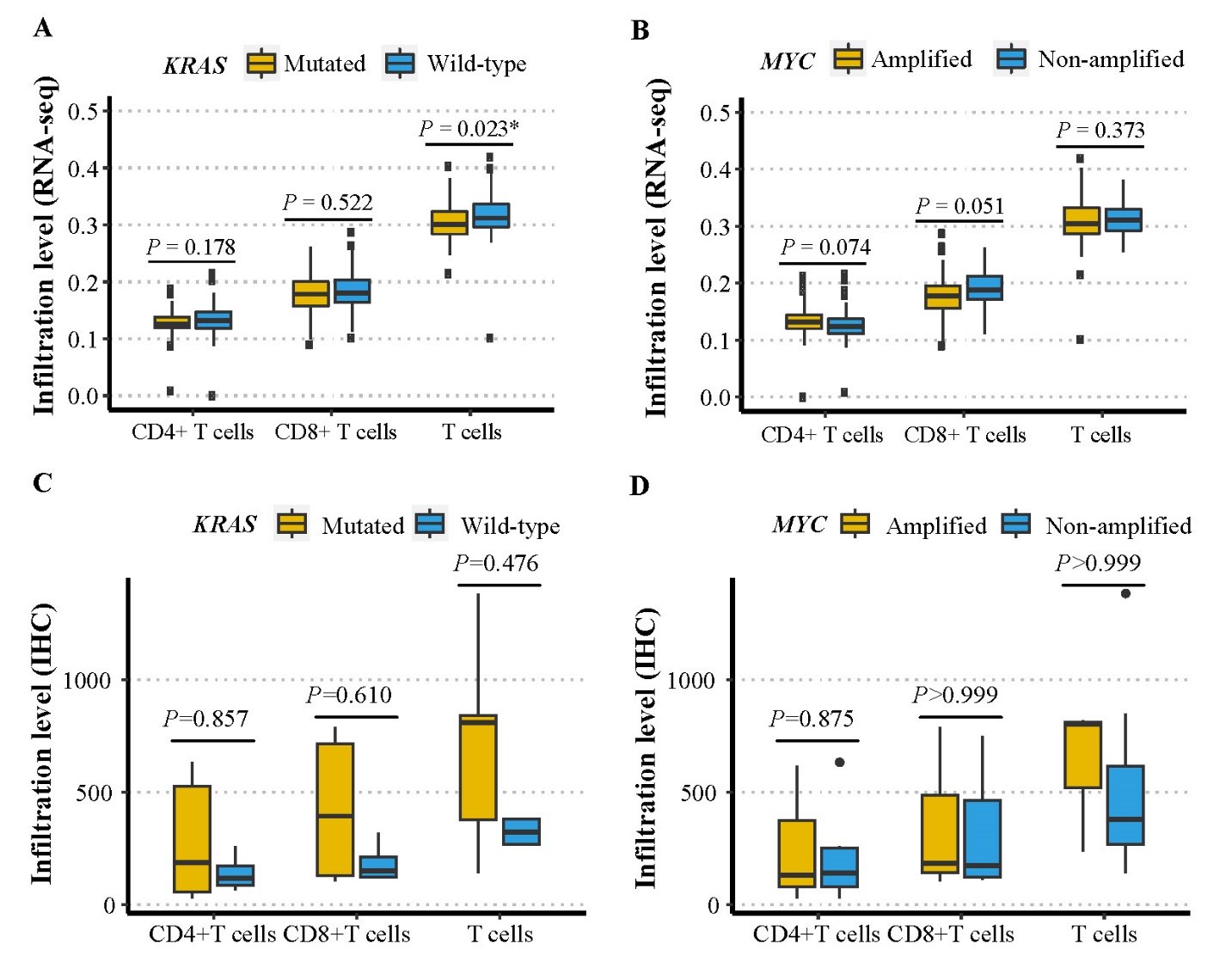


**Figure S3** The immune infiltration differences for patients with different mutational statuses. (**A, B**) Comparing *KRAS* mutations (**A**) or *MYC* amplification (**B**) status and RNA-seq-derived immune infiltration in 125 rectal cancer patients obtained from the TCGA database. (**C, D**) Comparing *KRAS* mutations (**C**) or *MYC* amplification (**D**) status and IHC-derived immune infiltration in 10 rectal cancer patients from our cohort. IHC, immunohistochemistry; RNA-seq, RNA sequencing.

**Figure S4** The association between pTNM stage and disease-free survival. Kaplan-Meier curve of disease-free survival in 55 LARC patients in strata of pTMN stages.
